# Deep learning enhances acute lymphoblastic leukemia diagnosis and classification using bone marrow images

**DOI:** 10.3389/fonc.2023.1330977

**Published:** 2023-12-06

**Authors:** Basel Elsayed, Mohamed Elhadary, Raghad Mohamed Elshoeibi, Amgad Mohamed Elshoeibi, Ahmed Badr, Omar Metwally, Raghad Alaa ElSherif, Mohamed Elsayed Salem, Fatima Khadadah, Awni Alshurafa, Deena Mudawi, Mohamed Yassin

**Affiliations:** ^1^ College of Medicine, Qatar University, Doha, Qatar; ^2^ Faculty of Medicine, Mansoura University, Mansoura, Egypt; ^3^ Faculty of Medicine, Zagazig University, Zagazig, Egypt; ^4^ Cancer Genetics Lab, Kuwait Cancer Control Centre, Kuwait City, Kuwait; ^5^ Department of Medical Oncology, National Center for Cancer Care and Research, Doha, Qatar

**Keywords:** acute lymphoblastic leukemia, bone marrow images, medical image analysis, deep learning, convolutional neural networks, diagnosis, classification

## Abstract

Acute lymphoblastic leukemia (ALL) poses a significant health challenge, particularly in pediatric cases, requiring precise and rapid diagnostic approaches. This comprehensive review explores the transformative capacity of deep learning (DL) in enhancing ALL diagnosis and classification, focusing on bone marrow image analysis. Examining ten studies conducted between 2013 and 2023 across various countries, including India, China, KSA, and Mexico, the synthesis underscores the adaptability and proficiency of DL methodologies in detecting leukemia. Innovative DL models, notably Convolutional Neural Networks (CNNs) with Cat-Boosting, XG-Boosting, and Transfer Learning techniques, demonstrate notable approaches. Some models achieve outstanding accuracy, with one CNN reaching 100% in cancer cell classification. The incorporation of novel algorithms like Cat-Swarm Optimization and specialized CNN architectures contributes to superior classification accuracy. Performance metrics highlight these achievements, with models consistently outperforming traditional diagnostic methods. For instance, a CNN with Cat-Boosting attains 100% accuracy, while others hover around 99%, showcasing DL models’ robustness in ALL diagnosis. Despite acknowledged challenges, such as the need for larger and more diverse datasets, these findings underscore DL’s transformative potential in reshaping leukemia diagnostics. The high numerical accuracies accentuate a promising trajectory toward more efficient and accurate ALL diagnosis in clinical settings, prompting ongoing research to address challenges and refine DL models for optimal clinical integration.

## 1 Introduction

Acute lymphoblastic leukemia (ALL) encompasses a range of lymphoid neoplasms that originate from precursor cells of both B-lineage and T-lineage cells (1). These neoplasms may primarily manifest as an extensive leukemic process involving both the bone marrow and peripheral blood, or they can display localized tissue infiltration with limited bone marrow involvement, termed lymphoblastic lymphoma (LBL) (2). Although ALL and LBLs exhibit distinct clinical features, they appear to represent a continuous biological spectrum. The current classification by the World Health Organization categorizes these conditions as B- or T-lymphoblastic leukemia/lymphoma (3). ALL is the most common pediatric malignancy, with pediatric ALL constituting approximately 80% of cases (4, 5). However, when it arises in adults, ALL takes on a particularly different clinical presentation. Notably, in the recent era of novel agents, not all cases of adult ALL have a poor prognosis; in fact, some individuals now experience good prognoses (6). In the United States, the estimated occurrence of ALL is about 1.6 cases per 100,000 individuals (7, 8). Research conducted among children has pinpointed genetic conditions that make a fraction of ALL cases more likely to occur including Down syndrome, Fanconi anemia, Bloom syndrome, and Ataxia Telangiectasia (9–11).

The initial phase of the diagnostic process for ALL, particularly to distinguish it from acute myeloid leukemia (AML) involves examining the bone marrow. This is crucial because ALL, as per its definition, invariably manifests with bone marrow participation (12, 13). Additional specialized tests are used to complement bone marrow evaluation such as peripheral blood smear (PBS) assessment and flowcytometric immunophenotyping (14, 15). However, bone marrow aspiration and biopsy remains the gold standard for ALL diagnostic confirmation, which provides a complete examination of cellular structure and appearance which could help indicate prognosis and evolution of the disease later on (16). While this approach allows for more precise classification and subtyping, it is an invasive process that can be painful, especially in pediatric patients, and getting appropriate samples can be difficult. Peripheral blood smears, on the other hand, require studying blood samples under a microscope to analyze blood cell morphology. Although they provide a rapid and non-invasive method of detecting blasts, their diagnostic depth may not be as extensive as bone marrow analysis.

Artificial intelligence (AI) and machine learning (ML) breakthroughs have sparked a revolution in medical image analysis and hematological diseases as previously explored by our group (17–21). Deep learning (DL) is a subset of ML that uses artificial neural networks to learn from data. Convolutional neural networks (CNNs) are one type of DL algorithm that has been particularly successful in image classification tasks (22). CNNs are designed to recognize patterns in images by using a series of convolutional layers that extract features from the input image. These features are then passed through a series of fully connected layers that classify the image based on the extracted features (22). CNNs have demonstrated exceptional ability in evaluating and interpreting medical images, including microscopic bone marrow images (23, 24).

In addition to the strides made in deep learning-based approaches, it is essential to acknowledge recent non-DL-based works that have contributed to the field of hematological disorder detection (25). Despite their contributions, these non-deep learning methods often face limitations in handling the complexity and variability present in hematological images. They may struggle to adapt to diverse morphologies and may require extensive manual tuning for optimal performance. DL methods, with their ability to automatically learn hierarchical features and patterns, offer a promising alternative that can potentially overcome some of these limitations, providing a more adaptive and robust solution for hematological disorder detection.

Although DL models in ALL diagnosis are widely studied, the focus has primarily been on peripheral blood smear (PBS) samples, neglecting the crucial bone marrow aspirates and biopsies, which are the gold standard for leukemia diagnosis. Recent reviews have also missed the majority of studies involving digital image analysis of microscopic bone marrow images (26, 27). Therefore, the goal of this review is to investigate the uses of DL in redefining ALL diagnosis and categorization using bone marrow images, possibly leading to the development of automated systems that assist healthcare personnel in making precise and timely ALL diagnoses. Performance metrics of several DL models and architectures in the detection and/or classification of ALL will be discussed. Furthermore, we will discuss the possible limitations and benefits of applying these models.

## 2 Materials and methods

### 2.1 Search strategy

We developed our search strategy on the 11th of June 2023 in the PubMed/MEDLINE database. To ensure a broad search strategy, we used many terms, such as ‘acute lymphoblastic leukemia’, ‘acute lymphocytic leukemia’, ‘acute lymphoid leukemia’, ‘ALL’, ‘artificial intelligence’, ‘machine learning’, ‘deep learning’, and ‘neural network’. The search was not restricted by language or time frame. The developed search strategy was transferred to Scopus, Embase, and Web of Science databases using the Polyglot translator (28). The studies were then transferred to EndNote X9, where duplicates were detected and omitted.

### 2.2 Eligibility criteria

The review will encompass studies that meet specific inclusion criteria: (1) utilization of human ALL samples, (2) publication in English, (3) employment of DL techniques for diagnosing/classifying ALL, (4) utilization of bone marrow samples, and (5) reporting of performance metrics. Studies not meeting these criteria will be excluded, ensuring a focused and relevant analysis.

### 2.3 Study selection and screening

After applying our search strategy to the mentioned databases, studies were transferred to EndNote X9, where duplicates were identified and removed. Remaining articles were uploaded to the Rayyan platform for additional screening (29). In Rayyan, titles and abstracts were screened for preliminary eligibility by two reviewers, and any discrepancies were settled by consensus. The whole texts of the papers that had been determined to be eligible were then acquired and independently double-screened for inclusion or exclusion using the mentioned criteria, with discrepancies resolved through screening by a third member if needed.

### 2.4 Data extraction

The data extraction process involved the extraction of pertinent information from included studies, comprising the last name of the primary author and the publication year, country of origin, dataset utilized, the targeted outcome under investigation, the applied validation methodologies, the employed models, and their corresponding performance metrics including accuracy, precision, sensitivity (recall), specificity, and F1 score. Furthermore, the strengths and limitations associated with each model were noted. Two investigators examined and obtained data from the eligible study independently. When they were unable to reach an agreement, they held a meeting with all team members. Only if it was agreed upon in the team meeting was the study included in the final review.

### 2.5 Aims

This review aims to provide an extensive examination of contemporary DL algorithms employed for the diagnosis and classification of ALL, with a specific emphasis on bone marrow samples. The principal objective entails a comprehensive evaluation of the performance of the diverse DL models featured in each study. Concurrently, a secondary aim involves the analysis of the relative merits and constraints of individual models in comparison to others.

## 3 Results

### 3.1 Study selection

**Figure 1:**
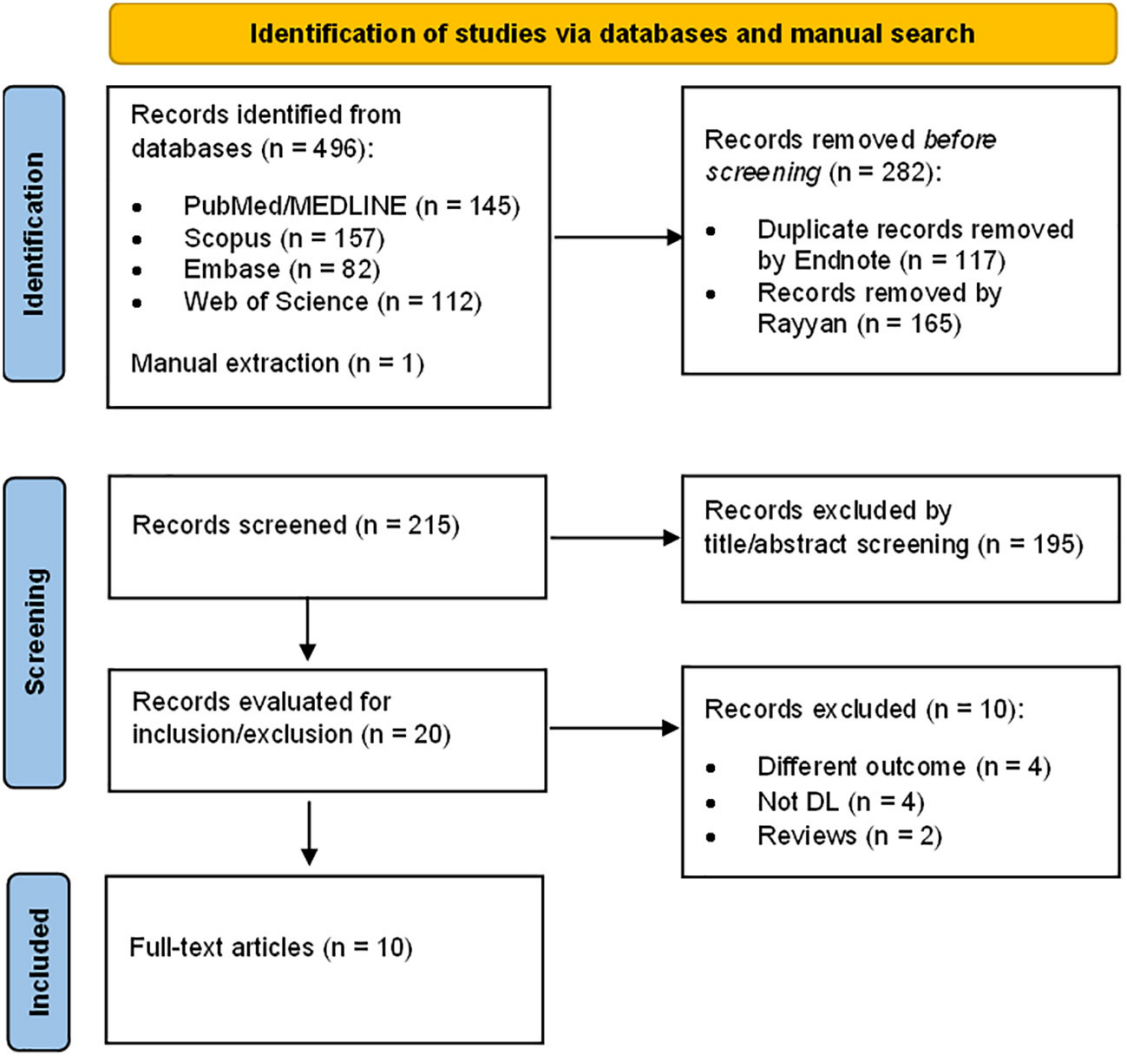
Schematic representation of the literature review process.

The PRISMA flow diagram, shown in **Figure 1**, depicts the process of selecting studies for this review. Initially, our database search yielded 496 results, with an additional article found through manual extraction. After removing 282 duplicates with EndNote and Rayyan, we evaluated the remaining 215 items based on their titles and abstracts. Through this screening process, we excluded 195 articles not eligible for further screening and were left with 20 for full-text screening. We retrieved and examined the complete texts of the 20 studies and based on a variety of reasons listed in **Figure 1**, we eliminated 10 more articles. Ultimately, 10 studies were included in our review.

### 3.2 Study characteristics and data collection

**Table 1** presents the attributes and data gathered from the studies included in this analysis. It evaluates the effectiveness of deep learning models implemented for the diagnosis and categorization of ALL through bone marrow imagery. For a more comprehensive understanding, the specific metrics for accuracy (ACC), precision (PRE), sensitivity (SEN), specificity (SPE), and F1-score are provided for each model. In summary, the studies covered were published between 2013 and 2023, predominantly originating from India (n = 5), China (n = 3), KSA (n = 1), and Mexico (n = 1). Among these, five studies utilized the SN-AM dataset, comprising microscopic bone marrow aspirate images from patients diagnosed with B-cell ALL and Multiple Myeloma (MM) (40).

**Table 1:** Performance of DL models in ALL diagnosis and classification using bone marrow images.

Authors (Year)	Country	Dataset & Sample Size	Validation (IV/EV)	Best Model(s)	ACC (%)	PRE (%)	SEN (%)	SPE (%)	F1 (%)
Devi et al. (2023) (30)	India	SN-AM dataset (B-ALL [90] and MM [100])	IV (Train-Test)	CNN (Convolutional Leaky RELU) with Cat-Boosting algorithm	100	100	99.9		100
				CNN (Convolutional Leaky RELU) with XG-Boosting algorithm	97.12	98.5	99		97.2
Duggal et al. (2017) (31)	India	BM samples (ALL [4469], healthy [4469])	IV (5-fold CV)	Texture-CNN with an additional SD-Layer	93.20				93.08
				CNN (AlexNet) with an additional SD-Layer	88.5				88.32
Huang et al. (2020) (32)	China	BM samples (ALL [23], AML [53], CML [10], healthy [18])	IV (Train-Test)	CNN (DenseNet121) with Transfer Learning technique	99				
Ikechukwu et al. (2022) (33)	India	SN-AM dataset (B-ALL [90] and MM [100])	IV (Train-Test)	CNN (i-Net)	99.18	99.30	99.18		99.19
Kavitha et al. (2022) (34)	India	SN-AM dataset (B-ALL [90] and MM [100])	IV (Train-Test)	CNN with Cat-Swarm Optimization	99.6	99.2	99.5	99.3	99.89
Kumar et al. (2020) (35)	India	SN-AM dataset (B-ALL [90] and MM [100])	IV (Train-Test)	Dense CNN (DCNN)	97.25	100	93.97	95.19	96.89
Ordaz-Gutierrez et al. (2013) (36)	Mexico	BM samples (ALL [118], healthy [62])	IV (Train-Test)	Hybrid of Fuzzy Logic and RBFNN	96.7		98.00	91.00	
Rehman et al. (2018) (37)	KSA	BM samples (ALL L1 [100], ALL L2 [100], ALL L3 [30], healthy [100])	IV (10-fold CV)	CNN (AlexNet)	97.78				
Yang et al. (2023) (38)	China	BM samples (ALL [306], AML [500], CML [162], healthy [291])	IV (Train-Test) EV (SN-AM)	Hybrid of CNN and ViT (MobileViTv2) with MultiPathGAN	96 (IV) 99.72 (EV)				
Zhou et al. (2021) (39)	China	AI-cell database^1^ (ALL [24], AML [25])	IV (Train-Test)^1^ EV (BM samples)	Ensemble of CNNs (ResNext101_32x8d, ResNext50_32x4d, and ResNet50)	89 (EV)		86 (EV)	95 (EV)	

ACC, Accuracy; PRE, Precision; SEN, Sensitivity SPE, Specificity; BM, Bone Marrow; ALL, Acute Lymphoblastic Leukemia; MM, Multiple Myeloma; AML, Acute Myeloid Leukemia; CML, Chronic Myeloid Leukemia IV, Internal Validation; EV, External Validation; CNN, Convolutional Neural Network; SD-Layer, Stain Deconvolutional Layer; ViT, Vision Transformer; RBFNN, Radial basis function neural network.

^1^Performance metrics of internally validated WBC classifier model: Accuracy: 82.93%, Precision: 85.67%, F1 score: 82.93%, AUC: 98.70%.

**Figure 2:**
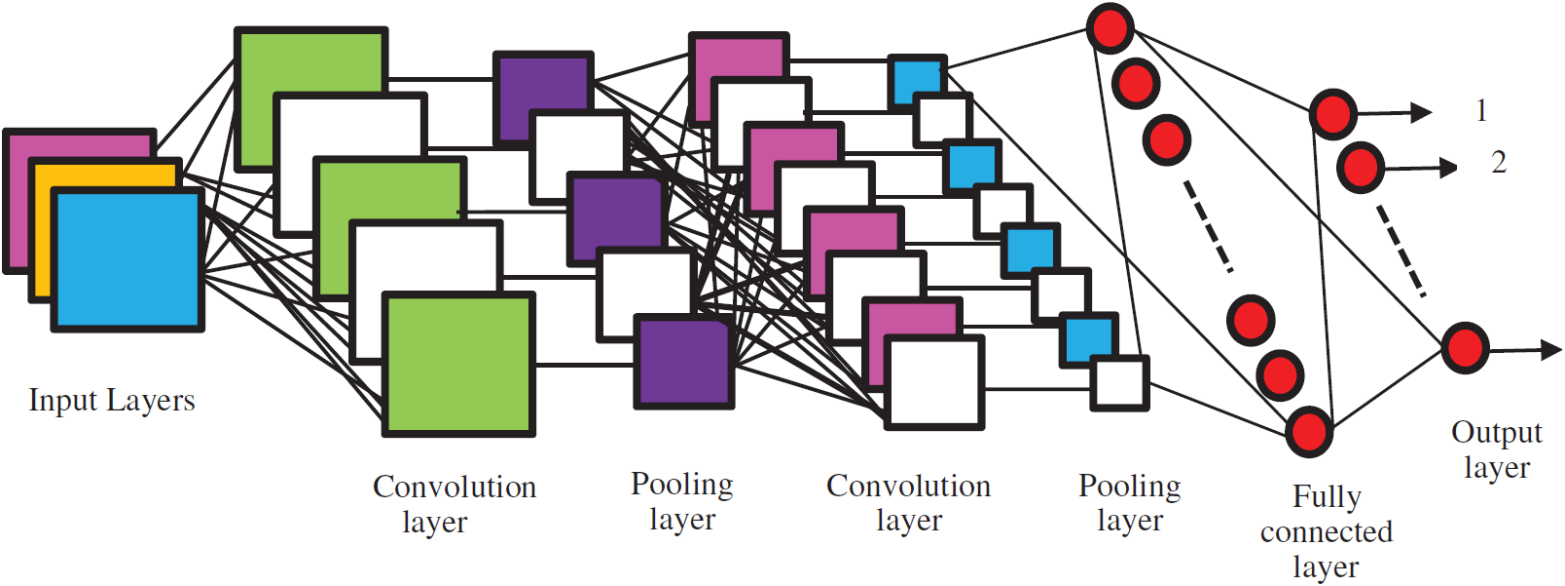
Convolutional neural network and its layers, adapted from Kavitha et al.

Notably, Yang et al. employed the SN-AM dataset in conjunction with the ALL-IDB1 database of peripheral blood smear images for external validation (38). Their model’s training and testing employed bone marrow samples from patients representing diverse leukemia families and subtypes. Zhou et al. introduced a novel “AI-cell platform” database for white blood cell (WBC) classification using bone marrow images, externally validating their model on authentic clinical samples of ALL and acute myeloid leukemia (AML) (39). The remaining four studies retrospectively sourced bone marrow aspirate images from hospital records. In terms of validation methods, only two studies conducted both external and internal validation, specifically utilizing a Train-Test Split approach. The remaining eight studies solely relied on internal validation via Train-Test Split (6) and k-fold cross-validation (2). Most of the studies employed CNNs as their classifier model, incorporating various layers, optimizations, and supplementary algorithms. The architecture of CNNs, as adapted from Kavitha et al, is depicted in **Figure 2**. A singular study deviated by utilizing radial basis function neural networks with a fuzzy logic algorithm in place of CNNs. The prevalence of CNNs, transfer learning, gradient boosting algorithms, and other elements typically associated with supervised learning tasks underscores these models’ intent for tasks involving labeled training data. Most models exhibited remarkable performance in their designated tasks. Notably, the models achieved high accuracy, with some reaching 100%, demonstrating the robustness of DL in ALL diagnosis.

**Table 2** provides an overview of the strengths and limitations of each DL model discussed in the review. Each model’s outcomes, including feature selection, boosting algorithms, and optimized hyperparameters, are highlighted as strengths. However, limitations such as the lack of external validation, dependency on image quality, and computational complexity are also discussed. These strengths and limitations are crucial in evaluating the practical applicability and potential challenges associated with each DL model. For instance, the discussion on the lack of external validation emphasizes the need for further validation on diverse datasets to ensure the generalizability of the models. Moreover, the acknowledgment of computational complexities and interpretability issues provides insights into areas where improvements and future research could be directed.

**Table 2:** Strengths and limitations of DL models reported.

Authors (Year)	Outcome	Strengths	Limitations
Devi et al. (2023) (30)	Classification of B-ALL and MM using CNNs with boosting algorithms.	Feature selection Use of boosting algorithms Reduced pre-processing	Lack of external validation Limited dataset Complex segmentation
Duggal et al. (2017) (31)	Differentiating malignant WBCs from normal WBCs using a CNN with a SD-Layer.	Stain deconvolution Minimal additional parameters Generalization potential	Lack of external validation Stain variation challenges Complexity for large datasets
Huang et al. (2020) (32)	Distinguishing between different types of leukemia using a CNN with transfer learning.	Multiple leukemia types Feasibility for small datasets Minimize need for segmentation	Lack of external validation Misclassification of leukemias Limited interpretability
Ikechukwu et al. (2022) (33)	Detection and classification of B-ALL and MM using a CNN with tuned hyperparameters.	Simplified architecture Feature selection capability Real-time applicability	Lack of external validation Limited dataset Limited interpretability
Kavitha et al. (2022) (34)	Detection and classification of B-ALL and MM using a CNN with Cat-Swarm Optimization algorithms.	Outperforms ML models Optimized hyperparameters Real-time applicability	Lack of external validation Limited dataset Computational complexity
Kumar et al. (2020) (35)	Detection and classification of B-ALL and MM using a Dense CNN with fewer parameters.	Outperforms ML models Feature extraction capability Real-world application	Lack of external validation Limited dataset Limited interpretability
Ordaz-Gutierrez et al. (2013) (36)	Diagnosis of ALL using fuzzy logic algorithm and Radial basis function neural network.	Handling of ambiguity Thorough cellular assessment Detection of ALL at early stages	Lack of external validation Dependency on image quality Specific cellular features
Rehman et al. (2018) (37)	Detection and classification of ALL and ALL subtypes (L1, L2. L3) using CNN.	Rapid diagnosis Robust segmentation Assist pathologists	Lack of external validation Limited dataset Dependency on image quality
Yang et al. (2023) (38)	Diagnosis and classification of leukemias using MobileViTv2 classifier and MultiPathGAN.	External validation Lightweight hybrid network Flexibility and adaptability	Sensitivity to data quality Lack of real-world scenarios
Zhou et al. (2021) (39)	Diagnosis of ALL in real clinical scenarios using an ensemble of CNN models.	Real-world & external validation Large dataset Mimics hematologist workflow	Single-center data Prospective validation needed

### 3.3 Specialized CNN designs

Section 3.3 is a discussion of studies that focused on developing specialized CNN designs, including the integration of specific enhancements, additional layers, and boosting algorithms to increase classification accuracy. It will be divided into studies using the SN-AM dataset and studies using retrospectively collected hospital bone marrow samples.

#### 3.3.1 B-ALL and MM classification using the SN-AM dataset

B-ALL and MM are hematologic malignancies that arise from various stages of B-cell development. Despite their evident clinical and pathological distinctions, certain resemblances exist in their morphological attributes and molecular characteristics, rendering their differentiation challenging. This subsection focuses on a series of studies that employ the SN-AM dataset, containing bone marrow images from patients with B-ALL and MM, to develop specialized CNN designs for accurate classification. In the four studies, the model was evaluated using internal validation through train-test split, partitioning data into training, validation, and testing sets with no external validation.

The article by Devi et al. addresses the segmentation and classification of white blood cancer cells within bone marrow microscopic images. The research methodology starts with data preprocessing, effectively eliminating dataset anomalies. Following that, dataset diversity and comprehensiveness are augmented through data augmentation techniques. The proposed model then utilizes the Convolutional Leaky RELU with CatBoost and XGBoost (CLR-CXG) algorithm for image segmentation and feature extraction, which are key processes for accurate classification. Binary classification is executed through CNN, accompanied by gradient boosting using CatBoost and XGBoost algorithms individually. The interaction between CNN and boosting algorithms mainly occurs in the classification phase. The features extracted by the CNN are used as inputs to the boosting algorithms, which refine the classification decision. This combination allows for a more accurate and efficient classification of blood cancer cells. This CLR-CXG approach aims to minimize bias and amplify accuracy in cancer cell classification, primarily discerning between B-ALL and MM. Internal validation is achieved by partitioning the dataset into train, test, and validate sets. CNNs play a pivotal role in image classification, recognizing important features within images through weight and bias assignment. To address challenges like input-output consistency and GPU expenses, the CLR-CXG model introduces modifications into the CNN architecture. A novel element is the incorporation of the Leaky RELU activation function, elevating the architecture’s capabilities. The results impeccably show that CatBoost and XGBoost algorithms enhance accuracy and computational efficiency. The CLRC algorithm achieves an impressive 100% accuracy, precision, and specificity in cancer cell classification, complemented by a sensitivity (recall) of 99.9% and an F1 score of 100. Meanwhile, CLRXG attains 97.12% accuracy, alongside precision, sensitivity (recall), and specificity values of 98.5%, 99%, and 97.2%, correspondingly. Despite these achievements, the article has many limitations including absence of information regarding resource allocation, memory usage, and energy efficiency.

Moreover, the study by Ikechukwu et al. introduces a novel deep CNN model named “i-Net” for classification of ALL using microscopic images. The proposed approach utilizes data from the SN-AM and ALL-IDB datasets, both sourced from the cancer imaging archive (TCIA) repository. Initially, augmentation balanced limited data. Data preprocessing involved grayscale conversion, contrast enhancement, and resizing. For segmentation, they used a UNet model with InceptionV2 architecture, while a custom CNN was designed for image classification. The authors employed two well-known pre-trained deep learning networks, ResNet-50 and VGG-19. However, they adapted the weights and learning parameters instead of using pre-existing ones. An upgraded CNN model, “i-Net,” was introduced, adding convolutional layers and fine-tuning hyperparameters for better classification accuracy. To prevent overfitting during training, the authors used data augmentation, dropout regularization, and batch normalization techniques. The proposed “i-Net” achieved 99.18% accuracy on the SN-AM dataset, surpassing ResNet-50 (84.5%) and VGG-19 (93.5%). The model’s generalization was tested, highlighting its potential for clinical decision support systems. Despite limitations due to computational constraints and a smaller dataset, the proposed “i-Net” model outperformed established models, showing promise for clinical use.

Furthermore, Kavitha et al. introduce a groundbreaking methodology for the diagnosis and classification of bone marrow cancers, with a particular focus on ALL and MM. The proposed model employs optimized deep CNNs utilizing a novel CAT (Cat Swarm Optimization) algorithm for hyperparameter tuning (41). The process involves three essential phases: data preparation, data augmentation, and classification. The data preparation phase involves capturing microscopic images from bone marrow aspirate slides, which are stained using the Jenner-Giemsa method. These raw images are then pre-processed to create a dataset that is utilized for both training and testing purposes. Data augmentation techniques are employed to alleviate overfitting concerns and augment the model’s ability to generalize. The architecture of the CNN incorporates convolutional layers for feature extraction, pooling layers for dimension reduction, and fully connected layers for accurate classification. The introduction of the CAT algorithm further enhances the model’s overall performance by drawing inspiration from the behaviours of cats, combining seeking and tracing modes to effectively optimize the network’s parameters. The evaluation of the proposed approach is conducted using the SN-AM dataset. The results showcase remarkable achievements, with an outstanding accuracy of 99.6% attained in accurately predicting ALL. This performance surpasses that of pre-trained deep learning models, such as AlexNets, VGG-16 Nets, and U-Nets. The proposed model’s superiority is further substantiated through comprehensive comparisons with other machine learning methodologies, including support vector machines, random forest, and naïve bayes.

**Figure 3:**
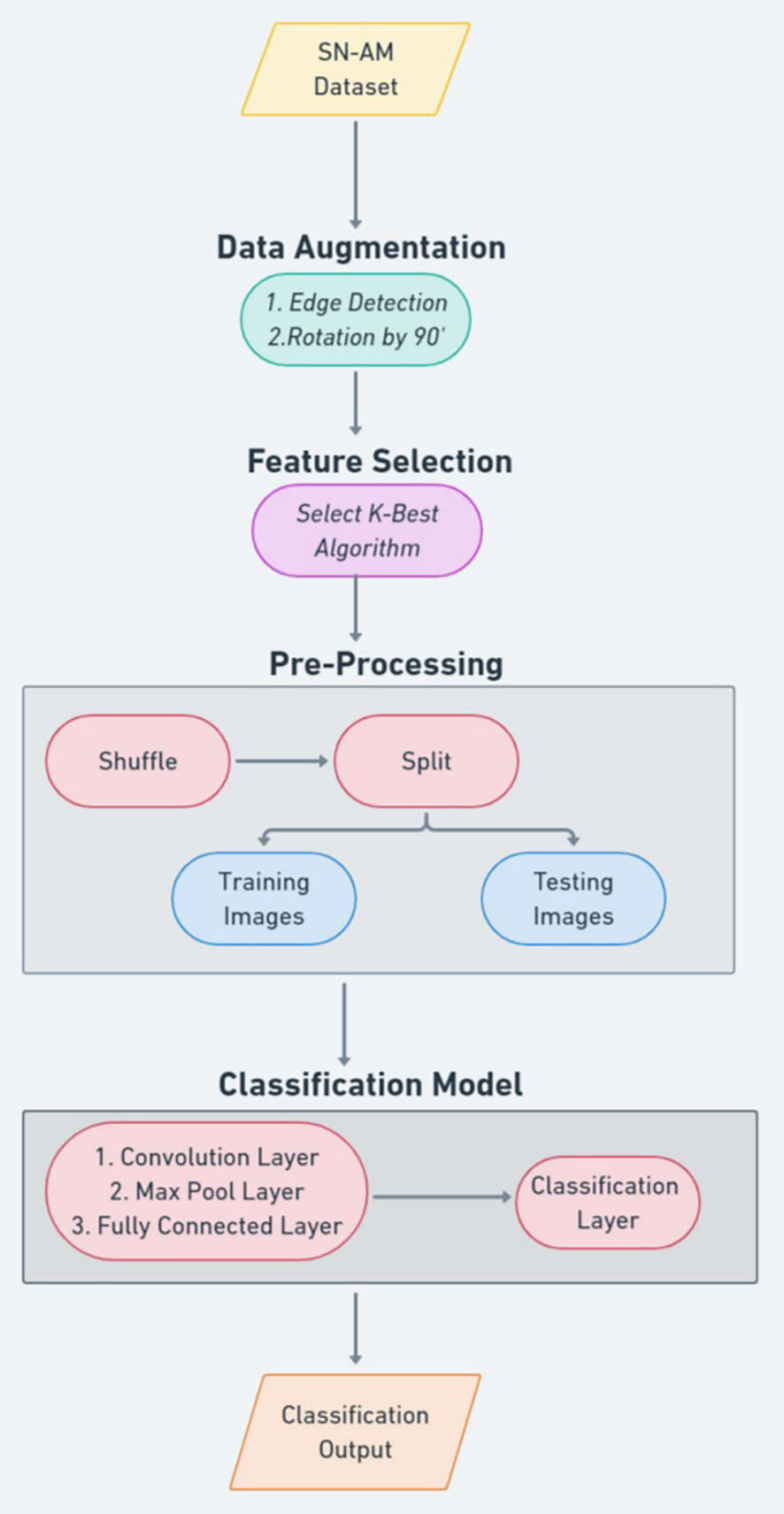
Kumar et al.’s proposed convolutional neural network-based methodology.

Lastly, the study conducted by Kumar et al. introduces a robust mechanism for classifying B-ALL and MM using CNNs. The study leverages deep learning techniques to automate the classification process, eliminating errors associated with manual assessment. The model is trained on cell images, undergoing preprocessing and feature extraction. It employs a dense convolutional neural network (DCNN) framework for classification, depicted in **Figure 3**, and achieved an impressive overall accuracy of 97.2%. Notably, the model demonstrates exceptional precision, sensitivity, specificity, and F1 score, with a precision of 100%, sensitivity of 93.97%, specificity of 95.19%, and an F1 score of 96.89%. The CNN architecture comprises convolution, max-pooling, and fully connected layers. Data augmentation techniques enhance generalization, while feature selection relies on the Chi-square test. Training utilizes an Adam optimizer with a sigmoid cross-entropy loss function and a learning rate of 0.01. Comparisons with machine learning methods and transferred learning models like VGG-16 were conducted. Random Forests achieved an accuracy of 96.83% on the dataset. However, the proposed CNN model significantly outperforms these approaches, boasting higher precision, sensitivity, specificity, and F1 score. Its capacity to extract features directly from images, coupled with adaptability across datasets, underscores its advantages. Although acknowledging limitations stemming from dataset size, the study underscores the potential of the proposed model as a reliable tool for diagnosing bone marrow blood cancers.

#### 3.3.2 ALL diagnosis using retrospectively collected hospital bone marrow samples

This subsection focuses on another set of studies that address the diagnosis of ALL through the analysis of retrospectively collected bone marrow samples from hospital patients. These studies emphasize the importance of accurate and efficient diagnosis for different leukemia subtypes.

The article by Duggal et al. presents an innovative convergence of deep learning techniques and stain deconvolution in the domain of medical image analysis. While CNNs have proven successful in medical imaging, the authors highlight a crucial limitation: CNNs primarily function in the RGB color space, potentially missing the nuanced tissue-stain interactions crucial for precise diagnostics. To address this, the study introduces the stain deconvolution layer (SD-Layer). Positioned at the forefront of CNN architectures, this layer operates in the optical density (OD) color space. Beer-Lambert’s law is employed to convert RGB microscopic images into the OD space, revealing pixel stain quantities that hold key diagnostic information. The SD-Layer operates on two fronts: converting RGB to OD space and using backpropagation to derive optimal stain basis vectors for diverse cell types. OD images are then deconvolved with these vectors, providing tissue-specific stain absorption quantities as input for downstream CNN layers. The study focuses on differentiating malignant WBCs from normal ones in cancer detection, particularly ALL. Texture-CNN and CNN (AlexNet) are evaluated using the SD-Layer in two modes: frozen (fixed stain vectors) and trainable (refined vectors). Impressively, the SD-Layer, initialized with stain basis vectors from SVD of the reference image, notably enhances classification accuracy for both architectures. This enhancement is attributed not to model capacity but to the biologically meaningful image representation the SD-Layer offers. With a well-structured dataset of around 9000 cell nuclei, balanced between normal and malignant cells and stained with Jenner-Giemsa, the study’s robustness is underscored. Rigorous training and augmentation techniques yield high performances on 5-fold cross-validation accuracy in distinguishing malignant from normal WBCs. The Texture-CNN achieves 93.20% accuracy and 93.08% F1 score with an additional SD-Layer, while CNN (AlexNet) achieves 88.5% accuracy and 88.32% F1 score with an additional SD-Layer. SD-Layer bridges RGB limitations, leveraging the OD space to capture crucial diagnostic insights. As medical imaging evolves, this study paves the way for harnessing stain quantities to enhance classification accuracy and diagnostic efficacy across diverse scenarios.

**Figure 4:**
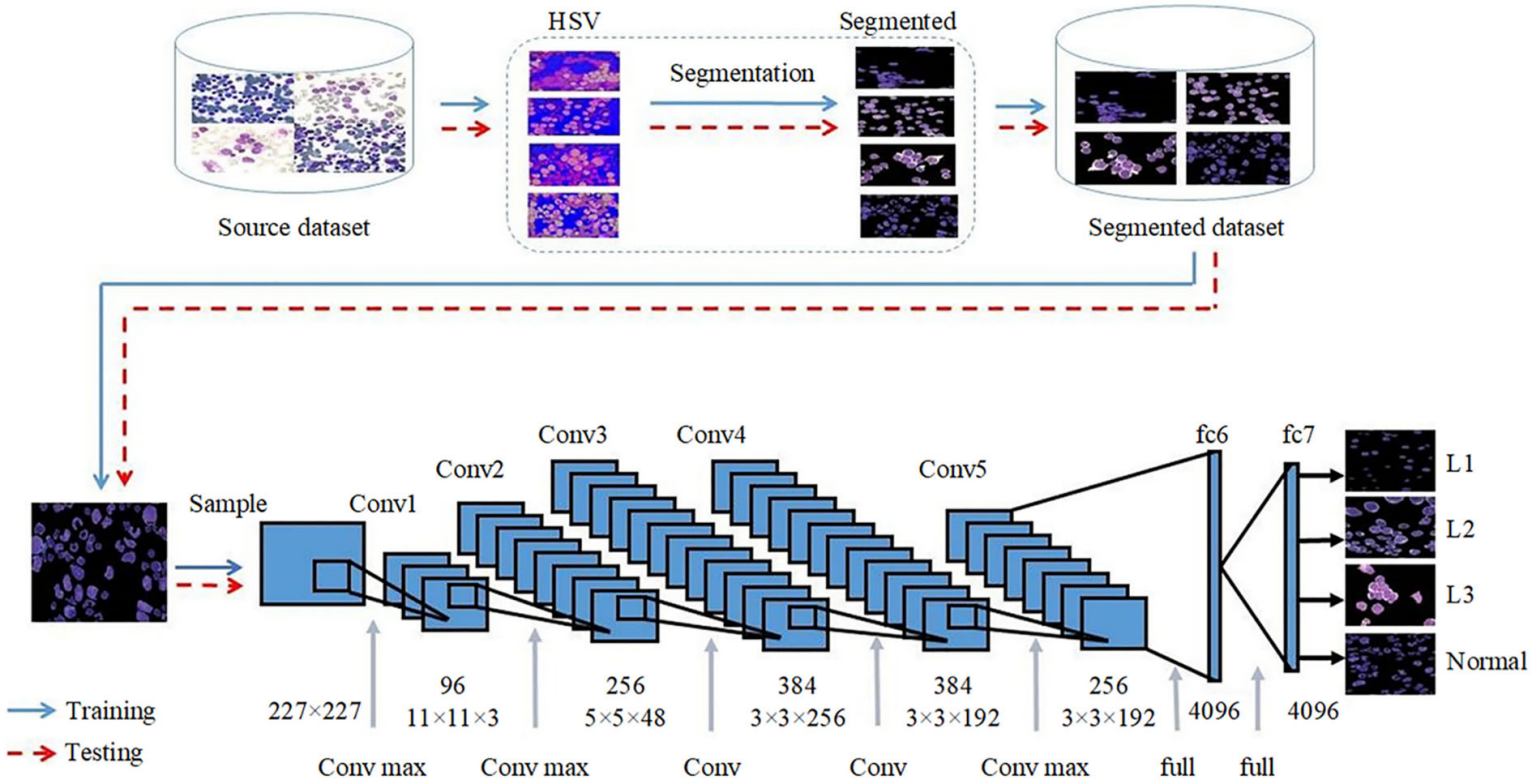
Rehman et al.’s proposed convolutional neural network-based methodology.

Moreover, Rehman et al., proposes a computer-aided system that combines image processing and deep learning to improve ALL diagnosis accuracy, depicted in **Figure 4**. The study focuses on classifying ALL into its subtypes and distinguishing reactive bone marrow (normal) using stained bone marrow images. The authors collect a dataset of bone marrow images from patients with ALL and normal cases. The images are captured using a digital microscope and processed to segment the regions of interest. A novel segmentation technique based on thresholding is introduced, followed by the application of CNNs for classification. The dataset is split into training and testing sets to train the CNN model. The researchers utilize the AlexNet architecture with transfer learning to fine-tune the model to the new data. To assess the effectiveness of their approach, the authors perform experiments and compare the results with other classification methods such as naïve Bayesian, K-nearest neighbor, and support vector machine. The proposed method achieves an impressive accuracy of 97.78% on the test dataset. The classification accuracy is plotted against the number of iterations, demonstrating that higher accuracy can be achieved with more epochs and a lower learning rate. The training time is also noted, with the proposed architecture taking approximately 163.63 seconds for 20 epochs. The authors highlight the significance of their work, as it provides an automated solution for accurate ALL diagnosis and classification. By employing DL techniques, the proposed system improves the accuracy of classification, which could significantly assist hematologists and pathologists in their diagnostic processes. Despite the promising results, this study does have limitations. The dataset size might impact the generalizability of the model, and external validation on larger datasets is necessary.

Lastly, Huang et al.’s study addresses leukemia classification and diagnosis through bone marrow cell morphology, employing CNNs alongside transfer learning. Traditional manual microscopy for leukemia diagnosis is subjective and error-prone, motivating an automated, precise approach. Their proposed method utilizes CNNs for identifying AML, ALL, and chronic myelocytic leukemia (CML). The researchers obtained microscopy images from healthy subjects and leukemia patients, implementing preprocessing techniques like perfect reflection and adaptive filtering to enhance quality and reduce background noise. They employed three CNN architectures for classification models on both raw and preprocessed datasets: Inception-V3, ResNet50, and DenseNet121. Transfer learning was leveraged to optimize model performance by extracting features or fine-tuning pre-trained models. In line with internal validation practices, the dataset is divided into a training set (991 samples) and a prediction set (331 samples) using a 3:1 ratio. The training set is utilized to train the models, while the prediction set serves as unseen data for testing the model’s generalization capability. DenseNet121 excelled among the CNN architectures, consistently achieving superior performance. Transfer learning notably expedited model convergence, significantly boosting accuracy. The study’s outcomes indicate that the DenseNet121 model on the preprocessed dataset garnered the highest accuracy at 74.8%. After transfer learning, its accuracy surged to 95.3%, a notable 20.5% improvement. The model exhibited accuracy rates of 90% for normal samples, 99% for ALL, 97% for CML, and 95% for AML, demonstrating efficacy in classifying various leukemia types. Nonetheless, the model faced challenges distinguishing immature granulocytes and lymphocytes, affecting AML classification accuracy. Its adaptability to rare leukemia types remains to be explored. Huang et al.’s study contributes a rapid, accurate, and objective method for leukemia diagnosis by merging CNNs with transfer learning. The combination overcomes the limitations of manual methods, catering to efficient, precise medical imaging despite small sample sizes. Though it confronts challenges, like distinguishing specific cell types, the study offers a promising path towards enhancing leukemia diagnosis and classification.

### 3.4 Ensemble and hybrid designs

In this section, we explore studies that utilize ensemble techniques and hybrid approaches, combining multiple models to enhance diagnosis accuracy. The subsequent subsections present the findings from these studies and provide insights into their contributions to the field.

#### 3.4.1 ALL Diagnosis using a hybrid of fuzzy logic and radial basis function neural network

This subsection examines the work by Ordaz-Gutierrez et al., which introduces an algorithm for diagnosing ALL using a combination of robust fuzzy logic and radial basis function neural networks (RBFNN). The primary aim of this research is to develop a reliable method for diagnosing ALL, particularly in developing countries like Mexico, where laboratory resources and equipment might be limited. The algorithm leverages bone marrow aspirates to extract specific features related to the disease. The process begins with acquiring microscopic cell images, which are then converted to grayscale to eliminate unnecessary color information and reduce processing time. Histogram equalization enhances image contrast. The segmentation stage involves utilizing the Sobel edge detection and mathematical morphology algorithms to isolate cells from the images. Suitable mathematical expressions are utilized to analyze cell size, circularity, and nuclei-to-cytoplasm ratio, crucial for ALL diagnosis. The heart of the method lies in applying fuzzy logic, chosen for incorporating human knowledge and mathematical modeling. The algorithm determines if a cell has ALL based on computed features. Fuzzy membership values combine using algebraic expressions to generate a diagnosis variable that classifies cells. To enhance the algorithm, a radial basis function (RBF) neural network is introduced, improving classification accuracy. Trained on a dataset, the algorithm achieves high sensitivity (98.00%) and specificity (91.00%). The results of the proposed method are promising, outperforming comparative methods, showing superiority in detection rates. The potential for real-time diagnosis is highlighted due to efficient feature extraction and RBF’s computational speed.

#### 3.4.2 Diagnosis of leukemias using a hybrid of CNN and vision transformer

Here, we delve into the article by Yang et al. which presents a deep learning-based approach for diagnosing leukemias using bone marrow aspirates. The study collected 2033 microscopic images of bone marrow samples, encompassing images for 6 disease types and 1 healthy control, from two Chinese medical websites. These images were divided into training, validation, and test datasets. To address variations in staining styles, a novel method called “stain domain augmentation” was introduced using the MultiPathGAN model. This technique normalized stain styles and expanded the dataset. A lightweight hybrid model named MobileViTv2, combining strengths of CNNs and vision transformers (ViTs), was developed for disease classification. MobileViTv2 achieved an average accuracy of 94.28% on the test set, with the highest accuracy values (98%, 96%, and 96%) obtained for MM, ALL, and lymphoma, respectively. Patient-level prediction accuracy averaged 96.72%. The model outperformed both CNNs and ViTs despite using only 9.8 million parameters. Furthermore, MobileViTv2 was compared to other deep learning models, demonstrating its superiority. The model’s effectiveness was also externally validated on public datasets (ALL-IDB1 and SN-AM), achieving high accuracy values of 99.75% and 99.72%, respectively. This indicates its robust generalization ability. While the model shows promise, there are some limitations. The dataset size is relatively small, and efforts to collect more images from various scanning devices could enhance its performance. Additionally, the model’s application is primarily focused on diagnosing broad disease categories, not specific subtypes. Future research could explore finer disease subtype classification and optimization of the model.

#### 3.4.3 Leukemia diagnosis using ensemble CNN models in real clinical scenarios

**Figure 5:**
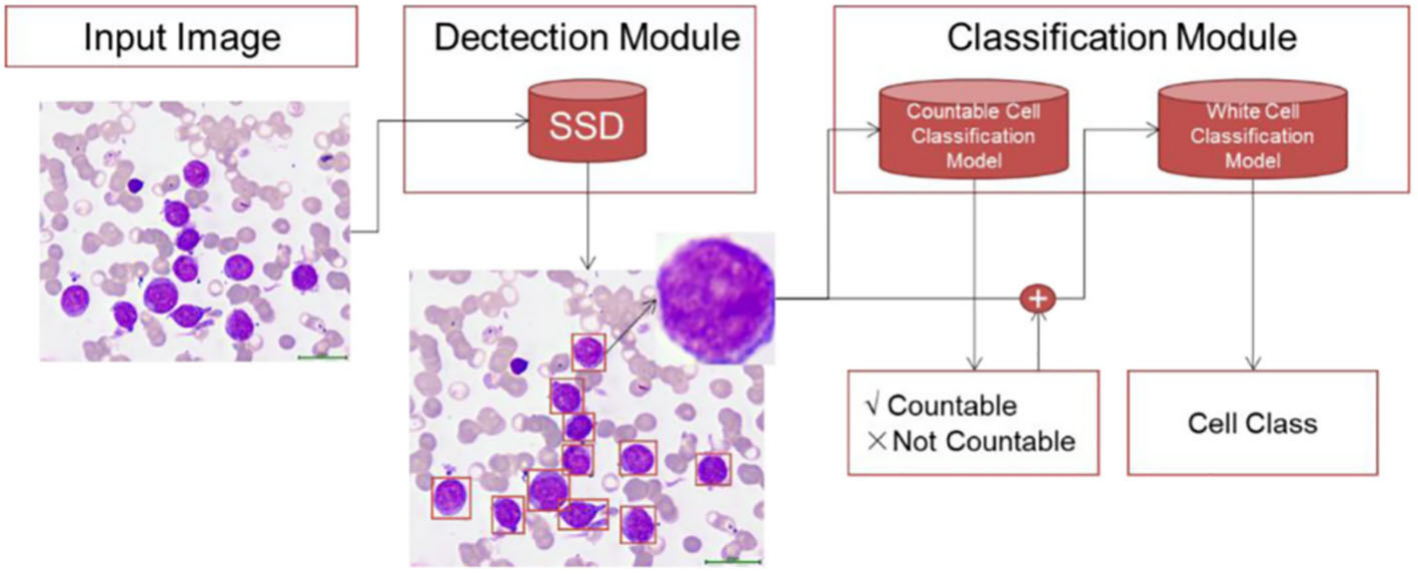
Zhou et al.’s proposed framework for white blood cell classification.

This subsection explores Zhou et al.’s study, which develops a deep learning-based system for leukemia diagnosis and evaluates it using real clinical scenarios. The subsection discusses the unique aspects of the system, its effectiveness in diagnosing different types of leukemia, and its practical utility in clinical settings. The researchers collected 1,732 bone marrow images, containing 27,184 cells, from children with leukemia in a dataset named “AI-cell platform”. This dataset was used to train a CNN architecture for the differential count of WBCs. Unlike prior approaches that preprocess images, this study used raw images without pre-processing. The developed system mimicked the process of hematologists by detecting and excluding uncountable and crushed cells, classifying remaining cells, and making diagnoses utilizing different configurations of ResNet and ResNeXt architectures for WBC detection and classification, as depicted in **Figure 5**. The ensemble of CNNs, comprising ResNeXt101_32x8d, ResNeXt50_32x4d, and ResNet50, emerged as the top-performing configuration. On internal validation using Train-Test Split, the ensemble model demonstrated very high performances for classifying WBCs (82.93% accuracy, 86.07% precision, and 82.02% F1 score). On external validation using real-world clinical samples of bone marrow, the system showed notable performance in diagnosing ALL (89% accuracy, 86% sensitivity, and 95% specificity). The validation results reveal significant insights into the ensemble’s performances and underscore its robustness and its potential for effectively diagnosing leukemia subtypes. Additionally, it accurately detected bone marrow metastasis of lymphoma and neuroblastoma (average accuracy of 82.93%). The system’s development was unique in using raw clinical images and replicating the hematologists’ workflow. The CNN differentiated crush cells, commonly excluded during manual counts, and demonstrated high accuracy across diverse WBC types. Furthermore, the system achieved successful ALL diagnosis in clinical practice, providing evidence of its practical utility. Comparison with existing studies revealed the uniqueness of this research in its broader variety of cell types, achieving high accuracy across complex clinical scenarios. Prior studies often focused on single-cell classification or employed pre-processed images, hindering real-world applications. While this study excelled in leukemia diagnosis, the limited dataset size for certain cell types posed challenges. However, the study’s innovative use of ensemble models mitigated this issue, enhancing overall accuracy.

## 4 Discussion

Recent advancements in medical image analysis have yielded remarkable progress in the automated detection and classification of acute leukemia, a critical hematological malignancy. Deep learning techniques have emerged as pivotal tools, demonstrating the potential to revolutionize diagnostic accuracy and efficiency. Key studies have explored acute leukemia detection and classification intricacies, addressing essential elements such as datasets, validation methodologies, and performance metrics.

### 4.1 Previous literature

The existing literature on AI-based acute lymphoblastic leukemia (ALL) classification, as discussed in systematic reviews by Das et al. (42) and Mustaqim et al. (43), reveals notable limitations that our review seeks to address. While these reviews have explored recent advancements in AI-based ALL classification, they primarily emphasize studies and datasets focused on peripheral blood samples. Although peripheral blood samples provide valuable insights, the gold standard for leukemia diagnosis has long been bone marrow samples, given their ability to offer a more comprehensive understanding of the disease’s characteristics. Bone marrow samples are particularly crucial for accurately distinguishing different leukemia subtypes. By including studies that utilize bone marrow samples in the context of AI-based ALL classification, our review fills this crucial gap in the literature, contributing to a more holistic understanding of advancements in leukemia diagnosis and emphasizing the significance of bone marrow analysis in achieving accurate and reliable results. Additionally, Alsalem et al.’s (44) comprehensive review on automated acute leukemia detection and classification complexities, while valuable, predominantly focuses on studies applying artificial intelligence to peripheral blood smear (PBS) samples (45–48). Similarly, Deshpande et al. (49) adopt an AI-centric approach, enhancing diagnostic accuracy through microscopic blood cell analysis. While these approaches contribute significantly to the field, they underscore the need for a more inclusive examination of bone marrow samples, as we address in our review. Furthermore, the literature showcases the effectiveness of deep learning models in distinguishing acute leukemia subtypes, such as the work by Anilkumar et al. (50) on automated B cell and T cell acute lymphoblastic leukemia differentiation using blood smear samples and Boldú et al.’s (51) introduction of ALNet for effective diagnosis of acute leukemia lineages using peripheral blood cell images. Moreover, Ouyang et al.’s (52) proposal of a convolutional neural network-based acute promyelocytic leukemia diagnosis highlights the versatility of deep learning across various subtypes. Laosai & Chamnongthai’s innovative approach using CD markers of blood cells for automated acute leukemia classification (15) adds another dimension to the literature. Notably, the significance of well-annotated datasets, such as ALL-IDB, SN-AM, C-NMC, and SDCT-AuxNet, in standardizing algorithm evaluation is acknowledged in the literature (53, 54). Finally, the literature emphasizes the importance of integrating real-world clinical scenarios and transfer learning to improve model robustness (55, 56), aspects that our review aims to further elucidate and emphasize in the context of bone marrow samples.

### 4.2 Specialized CNN designs

Several studies have been dedicated to the development of specialized CNN architectures, aiming to enhance the accuracy of classification. Devi et al. introduced the CLR-CXG model, synergizing convolutional leaky rectified linear units (ReLU) with CatBoost and XGBoost boosting algorithms for cancer cell classification. This integration showcased promising results, underlining the potential of hybrid models. The “i-Net” model by Ikechukwu et al. effectively combined pre-trained deep learning networks, segmentation techniques, and data augmentation to achieve exceptional accuracy in segmenting and classifying acute lymphoblastic leukemia (ALL). Kavitha et al. contributed an optimized deep CNN architecture for diagnosing bone marrow cancers, leveraging the Cat Swarm optimization (CAT) algorithm for hyperparameter tuning. By focusing on precise segmentation and feature extraction using deep CNNs, this study demonstrated the power of specialized designs in achieving robust classification. Additionally, Kumar et al. emphasized the automatic detection of white blood cell cancers, specifically ALL and MM using CNNs. Their study highlighted the capacity of CNNs to discern pertinent features within images, effectively enhancing classification accuracy.

### 4.3 Hybrid and ensemble designs

Hybrid and ensemble methodologies also emerged as valuable avenues for leukemia diagnosis. Ordaz-Gutierrez et al. introduced a practical hybrid approach, uniting the RBFNN with the fuzzy logic algorithm for ALL diagnosis. This study’s emphasis on resource-constrained settings underscores the importance of accessible and effective models. Yang et al. ventured into hybrid modeling by integrating CNNs with ViTs to diagnose hematologic malignancies through bone marrow images. By incorporating stain domain augmentation and hybrid modeling, this study showcased the potential of blending diverse deep learning techniques. Zhou et al. devised a deep learning-based system for leukemia diagnosis, employing an ensemble of multiple CNN models. The exceptional performance observed in classifying different white blood cell types and accurately diagnosing ALL in clinical scenarios exemplified the potential of ensemble techniques for practical medical applications.

### 4.4 Practical implications and limitations

The discussed studies offer a promising path for improving ALL diagnosis and classification through specialized CNN designs, hybrid models, and ensemble techniques. However, recognizing associated limitations is essential. One of the key limitations of the reviewed studies is the relatively small dataset sizes. While these studies demonstrate the potential of DL in ALL diagnosis, the limited data may raise concerns about the generalizability of the results. It is noteworthy that only two out of the ten reviewed studies employed external validation. This is a significant limitation, as relying solely on internal validation can lead to inflated performance metrics. To address these limitations, further efforts are required. This includes exploring larger datasets, refining segmentation techniques, and assessing clinical feasibility. It is imperative to develop a comprehensive evaluation framework that incorporates external validation and real-world clinical testing to enhance the robustness and generalizability of AI models for ALL diagnosis and classification. Moreover, considering the complexity of leukemia diagnosis, incorporating more complex samples, such as those with 10-15% blast in normal marrow, is vital for a thorough assessment of deep learning’s potential in distinguishing normal and malignant blasts.

### 4.5 Future considerations

Future research endeavors should consider the incorporation of molecular and genomic data into the analysis pipeline. Combining these data sources with image-based analyses can potentially provide a more holistic and accurate assessment of ALL cases. Furthermore, the development of AI models that can effectively integrate and interpret both image and molecular/genomic data represents an exciting avenue for future research in the field. By confronting limitations and pursuing the identified research agenda, the field can move into a new era of accurate, efficient, and accessible methods for diagnosing and classifying leukemia using bone marrow images. This advancement holds the potential to revolutionize clinical practices, enabling timely interventions and personalized treatment strategies. Moreover, establishing standardized protocols for external validation and benchmarking across different datasets and institutions will be instrumental in establishing the reliability and generalizability of deep learning models. Ultimately, as these future directions unfold, they will contribute to the ongoing refinement and deployment of AI-powered tools in the realm of ALL diagnosis and classification, improving patient outcomes and advancing the field of medical image analysis.

## 5 Conclusion

In conclusion, this review highlights the potential of deep learning models in enhancing acute lymphoblastic leukaemia diagnosis and classification. The proposed methodologies could revolutionize leukaemia diagnostics, providing accurate tools for early detection and treatment. Specialized CNN architectures, hybrid models, and ensemble techniques demonstrate the adaptability of deep learning in medical image analysis. However, limitations like small datasets and lack of external validation must be acknowledged. The reported high model performance metrics might be overestimated without robust validation. Future research should focus on refining and validating models, utilizing larger datasets, and conducting clinical feasibility studies. Collaborative efforts could integrate AI tools for precise leukaemia diagnosis, advancing patient care and reshaping medical imaging diagnostics.

## Author contributions

BE: Conceptualization, Methodology, Project administration, Writing – original draft, Writing – review & editing. ME: Methodology, Writing – original draft, Writing – review & editing. RME: Methodology, Writing – original draft, Writing – review & editing. AE: Methodology, Writing – original draft, Writing – review & editing. AB: Validation, Writing – original draft, Writing – review & editing. OM: Validation, Writing – original draft, Writing – review & editing. RAE: Validation, Writing – original draft, Writing – review & editing. MS: Validation, Writing – original draft, Writing – review & editing. FK: Writing – original draft, Writing – review & editing. AA: Writing – original draft, Writing – review & editing. DM: Writing – original draft, Writing – review & editing. MY: Conceptualization, Project administration, Supervision, Writing – original draft, Writing – review & editing.
